# Evidence for the Effectiveness of Remdesivir (GS-5734), a Nucleoside-Analog Antiviral Drug in the Inhibition of *I*_K(M)_ or *I*_K(DR)_ and in the Stimulation of *I*_MEP_

**DOI:** 10.3389/fphar.2020.01091

**Published:** 2020-07-21

**Authors:** Wei-Ting Chang, Ping-Yen Liu, Zi-Han Gao, Shih-Wei Lee, Wen-Kai Lee, Sheng-Nan Wu

**Affiliations:** ^1^ College of Medicine, Institute of Clinical Medicine, National Cheng Kung University, Tainan, Taiwan; ^2^ Division of Cardiovascular Medicine, Chi-Mei Medical Center, Tainan, Taiwan; ^3^ Department of Biotechnology, Southern Taiwan University of Science and Technology, Tainan, Taiwan; ^4^ Division of Cardiovascular Medicine, Department of Internal Medicine, College of Medicine, National Cheng Kung University Hospital, Tainan, Taiwan; ^5^ Department of Physiology, National Cheng Kung University Medical College, Tainan, Taiwan; ^6^ Institute of Basic Medical Sciences, National Cheng Kung University Medical College, Tainan, Taiwan; ^7^ Department of Medical Research, China Medical University Hospital, China Medical University, Taichung, Taiwan

**Keywords:** remdesivir (GS-5734), delayed-rectifier K^+^ current, electroporation-induced current, voltage hysteresis, pituitary cell, lymphocyte, M-type K^+^ current

## Abstract

Remdesivir (RDV, GS-5734), a broad-spectrum antiviral drug in the class of nucleotide analogs, has been particularly tailored for treatment of coronavirus infections. However, to which extent RDV is able to modify various types of membrane ion currents remains largely uncertain. In this study, we hence intended to explore the possible perturbations of RDV on ionic currents endogenous in pituitary GH_3_ cells and Jurkat T-lymphocytes. The whole-cell current recordings of ours disclosed that upon membrane depolarization in GH_3_ cells the exposure to RDV concentration-dependently depressed the peak or late components of *I*_K(DR)_ elicitation with effective IC_50_ values of 10.1 or 2.8 μM, respectively; meanwhile, the value of dissociation constant of RDV-induced blockage of *I*_K(DR)_ on the basis of the first-order reaction was yielded to be 3.04 μM. Upon the existence of RDV, the steady-state inactivation curve of *I*_K(DR)_ was established in the RDV presence; moreover, the recovery became slowed. However, RDV-induced blockage of *I*_K(DR)_ failed to be overcome by further addition of either α,β-methylene ATP or cyclopentyl-1,3-dipropylxanthine. The RDV addition also lessened the strength of M-type K^+^ current with the IC_50_ value of 2.5 μM. The magnitude of voltage hysteresis of *I*_K(M)_ elicited by long-lasting triangular ramp pulse was diminished by adding RDV. Membrane electroporation-induced current in response to large hyperpolarization was enhanced, with an EC_50_ value of 5.8 μM. Likewise, in Jurkat T-lymphocytes, adding RDV declined *I*_K(DR)_ amplitude concomitantly with the raised rate of current inactivation applied by step depolarization. Therefore, in terms of the RDV molecule, there appears to be an unintended activity of the prodrug on ion channels. Its inhibition of both *I*_K(DR)_ and *I*_K(M)_ occurring in a non-genomic fashion might provide additional but important mechanisms through which *in vivo* cellular functions are seriously perturbed.

## Introduction

Remdesivir (RDV, GS-5734), a broad-spectrum antiviral agent, is recognized as a mono-phosphoramidate prodrug of an adenosine analog that metabolizes into its active form GS-441524 which is a C-adenosine nucleoside analog (Warren et al., 2016; Lo et al., 2017; Sheahan et al., 2017; Tchesnokov et al., 2019; Gordon et al., 2020). This compound, a nucleotide-analog inhibitor of RNA-dependent RNA polymerase, is thought to be highly active against coronaviruses (CoVs), including MERS-Cov and SARS-CoV-2 (Lo et al., 2017; Sheahan et al., 2017; Agostini et al., 2018; Beigel et al., 2019; Brown et al., 2019; De Clercq, 2019; Ferren et al., 2019; Hoenen et al., 2019; Al-Tawfiq et al., 2020; de Wit et al., 2020; Dong et al., 2020; Khot and Nadkar, 2020; Ko et al., 2020; Lai et al., 2020; Li and De Clercq, 2020; Li Y. C. et al., 2020; Lu, 2020; Martinez, 2020; Morse et al., 2020; Sheahan et al., 2020; Wang et al., 2020). It has been recently recognized as a promising antiviral drug against an array of RNA viruses, predominantly through the targeting of the viral RNA dependent RNA polymerase. The active form GS-441524, into which RDV is metabolized, could inhibit cellular RNA polymerase to a lesser extent than viral polymerase (Agostini et al., 2018; Wang et al., 2020).

Recent studies have disclosed that RDV and chloroquine (or hydroxychloroquine) could be highly efficacious in control of the SARS-CoV-2 infection *in vitro* (Dong et al., 2020; Gao et al., 2020; Lai et al., 2020; Li and De Clercq, 2020; Wang et al., 2020). There are human studies of RDV efficacy for the treatment of SARS-CoV-2 infection (Beigel et al., 2020). However, none of the noticeable studies have been available with regard to the perturbing actions of RDV on membrane ion channels.

The voltage-gated K^+^ (K_V_) channels are essential in determining the membrane excitability in electrically excitable or non-excitable cells. Specifically, K_V_3 (KCNC) and K_V_2 (KCNB), two delayed-rectifier K^+^ channels, are widespread in different excitable cells such as endocrine cells (Lien and Jonas, 2003; Wang et al., 2008; Fletcher et al., 2018; Kuo et al., 2018; Lu et al., 2019; So et al., 2019). The causal link between the delayed-rectifier K^+^ current (*I*_K(DR)_) and K_V_3/K_V_2 channels has been previously disclosed (Yeung et al., 2005; Wang et al., 2008; Huang et al., 2013; Chang et al., 2019; Lu et al., 2019). The biophysical characteristics of K_V_3.1-K_V_3.2 channels, which are the dominant factors of *I*_K(DR)_ identified in pituitary tumor (GH_3_) cells (Chang et al., 2019; Lu et al., 2019; So et al., 2019), show a positively shifted voltage dependency as well as fast deactivation rate. However, whether and how RDV effects the adjustments on the amplitude and kinetic gating of above-stated types of K^+^ currents still requires investigations.

Furthermore, the KCNQ2, KCNQ3, and KCNQ5 genes have been noticed to encode the main subunits of K_V_7.2, K_V_7.3, and K_V_7.5 channels, respectively; and among them, the augmented activity produces the M-type K^+^ current (*I*_K(M)_), which is characterized by a slowly activating and deactivating property (Brown and Adams, 1980; Sankaranarayanan and Simasko, 1996; Wang et al., 1998; Selyanko et al., 1999; Shu et al., 2007; Lu et al., 2019; So et al., 2019; Yang et al., 2019). With growing recognition, targeting *I*_K(M)_ is regarded as a treatment of various neurologic diseases. How this compound acts on these types of K^+^ currents, however, remains largely uncertain.

Membrane electroporation (MEP) applies an external electrical field in situations where an increase in the electrical conductivity and permeability of the plasma membrane could be produced. Such maneuvers have been applied to the electrotransferation of membrane-impermeant molecules which include DNAs, anti-cancer drugs, and antibodies, into the internal milieu of cells (Liu et al., 2012; Napotnik and Miklavčič, 2018). Of notice, through applying an electrical field to the cells which exceed the electric capacity of surface membrane, it transiently and temporarily turns to be permeable and destabilized. Consequently, the molecules could readily and efficiently get into the cell (Wu et al., 2012; So et al., 2013; Napotnik and Miklavčič, 2018). In this scenario, to facilitate the uptake of antineoplastic or antiviral agents with difficulty in passing the cell membrane, MEP-induced current (*I*_MEP_) has been viewed as a novel therapeutic maneuver. However, as far as we are aware, none of studies have investigated whether the presence of RDV exerts any effects on *I*_MEP_.

For the considerations elaborated above, we attempted to inquire into the actions of RDV on different types of ionic currents (e.g., *I*_K(DR)_, *I*_K(M)_ and *I*_MEP_) in GH_3_ cells. Whether the *I*_K(DR)_ identified in Jurkat T-lymphocytes is subject to any modification by RDV was also tested. Noticeably, the present observations unveiled that, in GH_3_ cells, RDV is presumably not a prodrug, and that it is virtually effective in inhibiting *I*_K(DR)_ and *I*_K(M)_ with similar potency; however, it was noticed to increase the strength of *I*_MEP_. These actions demonstrated presently are prone to be acute in onset and will resultantly summate to affect electrical behaviors of different cell types. Findings from the present observations may conceivably contribute to its toxicological and pharmacological actions of RDV occurring *in vitro* or *in vivo*.

## Materials and Methods

### Chemicals, Drugs, and Solutions Used in This Study

Remdesivir (RDV, development code: GS-5734, C_27_H_35_N_6_O_8_P, 2-ethylbutyl (2*S*)-2-[[[(2*R*,3*S*,4*R*,5*R*)-5-(4-aminopyrrolo[2,1-f][1,2,4]triazin-7-yl)-5-cyano-3,4-dihydroxyoxolan-2-yl]methoxy-phenoxyphosphoryl]amino]propanoate) was from MedChemExpress (Bio-genesis Technologies, Taipei, Taiwan), while α,β-methylene ATP (AMPCPP), cyclopentyl-1,3-dipropylxanthine (DPCPX), ivabradine, nonactin, and tetrodotoxin were from Sigma-Aldrich (Merck, Taipei, Taiwan). Chorotoxin was a gift of Professor Woei-Jer Chuang (Department of Biochemistry, National Cheng Kung University Medical College, Tainan, Taiwan). In this study, we obtained the reagent water by using a Milli-Q Ultrapure Water Purification System (18.2 MΩ-cm) (Merck Millipore, Taipei, Taiwan) in all experiments.

The composition of bath solution (i.e., HEPES-buffered normal Tyrode’s solution) used in this study was (in mM): 136.5 NaCl, 5.4 KCl, 1.8 CaCl_2_, 0.53 MgCl_2_, 5.5 glucose, and 5.5 HEPES, adjusted with NaOH to pH 7.4. In attempts to check *I*_K(M)_ or *I*_K(erg)_, we substituted the bath solution for a high-K^+^, Ca^2+^-free solution (in mM): 130 KCl, 10 NaCl, 3 MgCl_2_, and 5 HEPES, adjusted with KOH to pH 7.4. To judge different types of K^+^ currents or *I*_MEP_, we backfilled the patch electrode with a solution (in mM): 130 K-aspartate, 20 KCl, 1 KH_2_PO_4_, 1 MgCl_2_, 0.1 EGTA, 3 Na_2_ATP, 0.1 Na_2_GTP, and 5 HEPES, adjusted with KOH to pH 7.2. To minimize any contamination of Cl^−^ currents, Cl^−^ ions inside the examined cell were mostly replaced with aspartate. In a different set of recordings for measuring the cation selectivity of ion channels, K^+^ ions inside the internal solution were replaced with NMDG^+^ ions.

### Cell Culture

GH_3_, originally acquired from the Bioresources Collection and Research Center ([BCRC-60015]; Hsinchu, Taiwan), were cultured in Ham’s F-12 medium added on with 15% (v/v) horse serum, 2.5% (v/v) fetal calf serum and 2 mM l-glutamine; while the Jurkat T cell line, a human T cell lymphoblast-like cell line (clone E6-1), was also from the Bioresource Collection and Research Center ([BCRC-60255]; HsinChu, Taiwan), and Jurkat T cells were grown in RPMI-1640 medium added on with 10% (v/v) fetal bovine serum. GH_3_ or Jurkat T cells were maintained at 37°C in a 95% air and 5% CO_2_ humidified atmosphere. The viability of these cells was often judged with the trypan blue dye-exclusion test. The electrical recordings were undertaken five or six days after cells had been cultured (60–80% confluence).

### Electrophysiological Studies

Briefly before the recordings, we harvested GH_3_ or Jurkat T cells and rapidly resuspended an aliquot of cell suspension to a custom-made cubicle mounted on the fixed stage of CKX-41 inverted microscope (Olympus; YuanLi, Kaohsiung, Taiwan). We the immersed cells at room temperature (20–25°C) in normal Tyrode’s solution, the composition of which has been described above in detail. We exploited either a P-97 Flaming/Brown horizontal puller (Sutter Instruments, Novato, CA) or a PP-83 vertical puller (Narishige; Taiwan Instrument, Taipei, Taiwan) to fabricate the recording pipette electrodes, which were made of Kimax-51 glass capillaries (Kimble; Dogger, New Taipei City, Taiwan), and we then fire-polished electrode tips with an MF-83 microforge (Narishige). The patch electrodes, in which different internal solutions were filled up, had a tip resistance of 3 to 5 MΩ. In this study, we undertook standard patch-clamp whole cell recordings at room temperature by applying either an RK-400 (Bio-Logic, Claix, France) or an Axopatch-200B patch-amplifier (Molecular Devices, Sunnyvale, CA). To measure whole-cell data, the junctional voltage between the pipette and bath solution was set as zero once the electrode was bathed but shortly before the giga-seal (>1 GΩ) formation. The details of data recordings and analyses achieved in the present work were described in **Supplementary Material**.

### Curve Fitting Procedures and Statistical Analyses

Curve parameter estimation was achieved either by a non-linear (e.g., Hill and Boltzmann equation or single-exponential function) or by linear fitting routine, in which the Solver add-in bundled with Excel 2013 (Microsoft, Redmond, WA) was undertaken. The experimental data in the present study are presented as the mean ± standard error of the mean (SEM), with sample sizes (n) representing the number of cells (e.g., GH_3_ or Jurkat T cells) collected. Student’s *t*-test and a one-way analysis of variance (ANOVA) were implemented and *post-hoc* Fisher’s least-significance difference test was applied for multiple comparison procedures. However, assuming that the results might violate the normality underlying ANOVA, the nonparametric Kruskal-Wallis test was thereafter performed. Statistical significance was regarded as *P* < 0.05.

## Results

### Inhibitory Effect of RDV on Depolarization-Evoked Delayed-Rectifier K^+^ Current (I_K(DR)_) Identified in GH_3_ Cells

**Figure 1:**
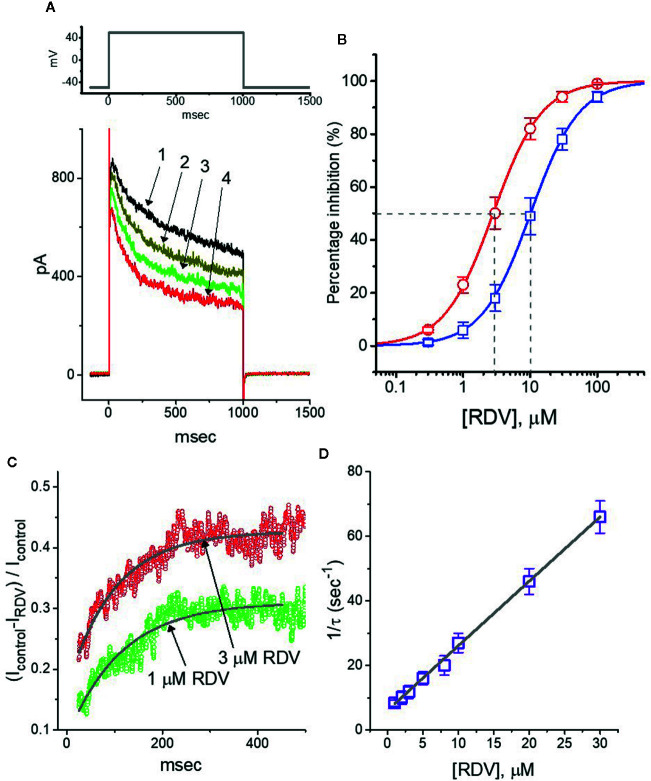
Effect of RDV on delayed-rectifier K^+^ current (*I*_K(DR)_) in pituitary GH_3_ cells. Cells were bathed in Ca^2+^-free, Tyrode’s solution and the recording electrode was backfilled up with K^+^-containing solution. **(A)** Superimposed *I*_K(DR)_ traces obtained in the control (**1**, i.e., RDV was not present), and during the exposure to 0.3 μM RDV (**2**), 1 μM RDV (**3**) or 3 μM RDV (**4**). The upper part is the voltage-clamp protocol applied to the cell. **(B)** Concentration-dependent inhibition of RDV on *I*_K(DR)_ amplitude measured at the beginning (**□**) and end (**○**) of depolarizing command potential (mean ± SEM; n=8 for each point). *I*_K(DR)_ amplitudes (i.e., transient or late component) in different RDV concentrations were taken at the beginning or end of depolarizing pulse for 1 sec from −50 to +50 mV. Continuous lines were well fitted with Hill equation as detailed in **Materials and Methods**. The IC_50_ value (as indicated by the vertical dashed line) measured in initial peak or late component of *I*_K(DR)_ was yielded to be 10.1 or 2.8 μM, respectively. **(C)** Relative block (i.e., (*I*_control_-*I*_RDV_)/*I*_control_) of *I*_K(DR)_ in the presence of 1 or 3 μM RDV. Smooth line in the presence of 1 or 3 μM RDV denotes the exponential fit with the time constant of 113.5 or 98.9 ms, respectively. **(D)** Relationship of the RDV concentration as a function of the rate constant (1/τ) (mean ± SEM; n=8 for each point). Based on minimal kinetic scheme described in **Materials and Methods**, the value of *k*_+1_^*^ or *k*_-1_ was estimated to be 2.01 s^−1^μM^−1^ and 6.12 s^−1^, respectively; and the *K*_D_ value (*k*_-1_/*k*_+1_^*^, i.e., dissociation constant) was resultantly yielded to be 3.04 μM.

In the first stage of experiments, we undertook the whole-cell configuration of standard patch-clamp technique applied to these cells. The experiments were conducted in cells bathed in Ca^2+^-free, Tyrode’s solution which contained 1 μM tetrodotoxin and 10 μM CdCl_2_, and we afterwards backfilled the recording electrode by utilizing K^+^-containing solution. Tetrodotoxin or CdCl_2_ in bathing solution was employed to block voltage-gated Na^+^ or Ca^2+^ currents, respectively. As depicted in **Figure 1A**, when we voltage-clamped the examined cells at −50 mV and then applied depolarizing command potential to +50 mV with a duration of 1 sec, the delayed-rectifier K^+^ current (*I*_K(DR)_) was able to be robustly evoked, as elaborated previously (Wang et al., 2008; Lu et al., 2019). Of notice, As exposed to RDV at various concentrations, the strength of *I*_K(DR)_ evoked by the corresponding depolarizing pulse was dose-dependently declined; however, the initial peak component of *I*_K(DR)_ was measurably decreased to a less extent as compared with the late component of the current. Depending on the modified Hill equation elaborated in *Materials and Methods* section, the IC_50_ value entailed for its inhibitory effects on initial peak or late components of *I*_K(DR)_ was yielded to be 10.1 or 2.8 μM, respectively (**Figure 1B**). As such, the experimental observations disclosed that during GH_3_-cell exposure to this compound, the late component of *I*_K(DR)_ by step depolarization applied from −50 to +50 mV was manifestly lessened to a greater extent than the initial peak component of the current.

Beyond the decreased strength of *I*_K(DR)_, as the cells exposed to different RDV concentrations, the increase of *I*_K(DR)_ inactivation relaxation responding to protracted depolarization was noticeably observed in a time-dependent manner. That is, the relaxation time course of *I*_K(DR)_ inactivation in the presence of this compound likely became strengthened, though the activation one of the current was unchanged. What is more, we measured the time constants of *I*_K(DR)_ inactivation in different RDV concentrations, as illustrated in **Figure 1C**, the time courses of relative block of *I*_K(DR)_, namely, (*I*_control_-*I*_RDV_)/*I*_control_, in the presence of different RDV concentrations were appropriately fitted to a single exponential process. Under minimal reaction scheme elaborated in the **Supplementary Material**, the estimated *K*_D_ value in the existence of RDV amounted to 3.06 μM (as indicated in **Figure 1D**), which is noticeably near the IC_50_ value warranted for RDV-mediated blockade of the late (or sustained) component of *I*_K(DR)_; however, it was noticeably lower than that for its depressant action on the initial peak component of the current.

### Inhibitory Effect of RDV on Averaged Current-Voltage (I-V) Relationship of I_K(DR)_

**Figure 2:**
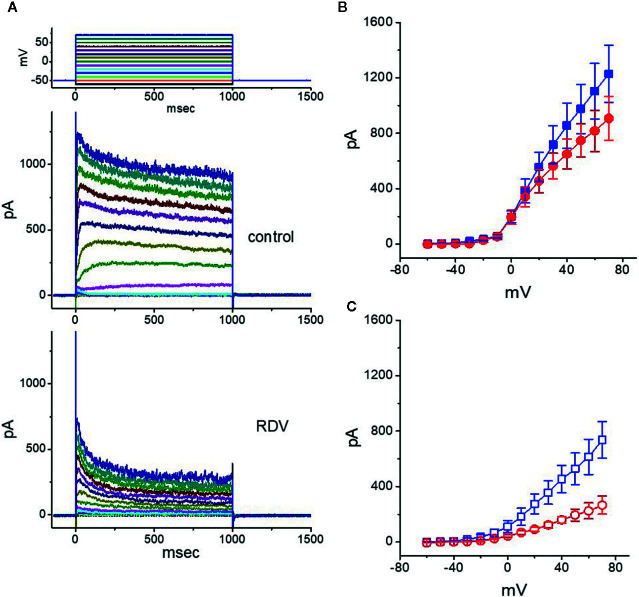
Effect of RDV on the current-voltage (*I-V*) relationship of *I*_K(DR)_ in GH_3_ cells. In the experiments on the elicitation of *I*_K(DR)_, the cell was maintained at −50 mV and 1-sec depolarizing command pulse to a series of voltage steps ranging between −60 to +70 mV in 10-mV increments was thereafter applied. **(A)** Representative *I*_K(DR)_ traces obtained in the control (upper) and during cell exposure to 10 μM RDV (lower). The uppermost part shows the voltage protocol delivered. In **(B, C)**, the averaged *I-V* relationships of *I*_K(DR)_ obtained in the absence (filled symbols) and presence (open symbols) of 10 μM RDV are illustrated, respectively (mean ± SEM; n=8 for each point). The data points in **(B, C)** were collected at the beginning (initial peak component, square symbols) or end (late component, circle symbols) of 1-sec depolarizing pulse.

In another separate series of measurements, we voltage-clamped at −50 mV and then delivered command voltage pulses from −60 to +70 mV in 10-mV increments with a duration of 1 sec to the examined cells. Under these experimental voltage protocols, a family of *I*_K(DR)_ could be robustly elicited and the currents were noticeably manifested by an outwardly rectifying property with a reversal potential of −74 ± 2 mV (n = 13) (Wang et al., 2008; Lu et al., 2019; So et al., 2019). Of notice, one minute after exposure to 10 μM RDV, the *I*_K(DR)_ strength was depressed particularly at the potentials ranging between −20 and +70 mV. **Figures 2A–C** depict the *I-V* relationships of *I*_K(DR)_ measured at the beginning (initial peak) and end (late or sustained) of each potential in the control and during cell exposure to 10 μM RDV. The magnitude for RDV-induced block of *I*_K(DR)_ measured at the end of depolarizing pulses (i.e., late *I*_K(DR)_) noticeably became greater than that achieved at the beginning of pulses (i.e., peak *I*_K(DR)_). For instance, at the level of +50 mV, RDV (10 μM) lessened the peak component of *I*_K(DR)_ by 46 ± 2% from 976 ± 178 to 527 ± 114 pA (n = 8, *P*<0.05). However, at the same level of voltage pulse, RDV at the same concentration distinctly declined the *I*_K(DR)_ amplitude attained at the end of depolarizing pulse by 74 ± 3% from 748 ± 121 to 194 ± 42 pA. After washout of RDV, the peak or late amplitude of *I*_K(DR)_ was back to 956 ± 168 or 732 ± 114 pA, respectively (n = 7). Meanwhile, from the current experimental conditions, the presence of 10 μM RDV significantly declined initial or late component of macroscopic *I*_K(DR)_ conductance (measured at the voltage from +30 to +70 mV) to 9.2 ± 0.2 or 3.5 ± 0.2 nS from the control values of 12.7 ± 0.6 or 8.5 ± 0.5 nS (n = 8), respectively. In consequence, the strength for RDV-induced block of late or steady-state *I*_K(DR)_ in dealing with step depolarizations was pronouncedly larger than that of instantaneous peak components of the current.

### Comparison Among the Effects of RDV, RDV Plus α,β-Methylene ATP (AMPCPP) and RDV Plus Cyclopentyl-1,3-Dipropylxanthine (DPCPX) on I_K(DR)_ Amplitude

**Figure 3:**
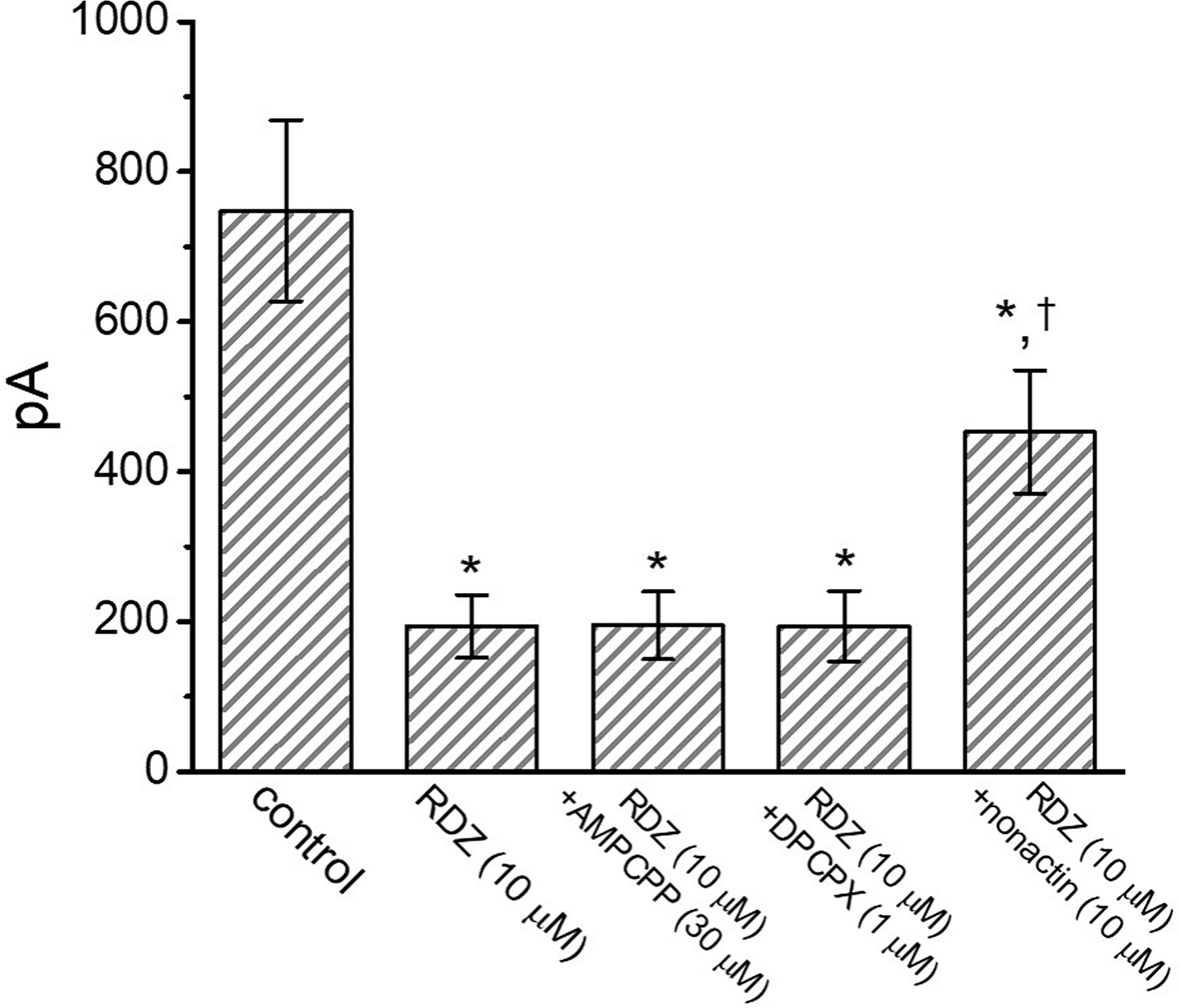
Comparisons among the effect of RDV, RDV plus α,β-methylene ATP (AMPCPP), RDV plus cyclopentyl-1,3-dipropylxanthine (DPCPX) and RDV plus nonactin on *I*_K(DR)_ amplitude in GH_3_ cells (mean ± SEM; n=8 for each bar). GH_3_ cells were bathed in Ca^2+^-free, Tyrode’s solution and the electrode was filled with K^+^-containing internal solution. Current amplitude from −50 mV depolarizing pulse to +50 mV depolarization with a duration of 1 sec was measured at the end of depolarizing command potential. In this set of experiments on RDZ plus each agent, the tested compound was subsequently added in the continued presence of RDV (10 μM). ^*^Significantly different from control (*P*<0.05).

It has been noticed that the binding of muscarinic or purinergic receptors to GH_3_ cells is likely to activate K^+^-channel activity through a G-protein modulation (Yatani et al., 1987). We hence examined whether adding AMPCPP or DPCPX, but still in the continued exposure to RDV, was able to adjust RDV-perturbed inhibition of *I*_K(DR)_ detected in GH_3_ cells. Of surprise, as depicted in **Figure 3**, neither further application of AMPCPP (30 μM) nor DPCPX (1 μM) effectively modified the inhibition of *I*_K(DR)_ produced by 10 μM RDV, in spite of the ability of RDV alone to depress *I*_K(DR)_ and to fasten current inactivation. AMPCPP, a non-degradable ATP analog, is previously reported to be a P_2X_-purinergic-receptor agonist, whereas DPCPX is an antagonist of adenosine A_1_ receptor (Wu et al., 1998). Alternatively, in the continued presence of 10 μM RDV, further application of 10 μM nonactin, known to be a K^+^ ionophore, could effectively reverse RDV-induced decrease of current amplitude. Therefore, RDV-perturbed strength of *I*_K(DR)_ observed in GH_3_ cells is most unlikely to be connected with its preferential binding to the purinergic or adenosine receptors, although the RDV molecule was thought to be a prodrug of an adenosine nucleoside analog (Lo et al., 2017; Brown et al., 2019; Tchesnokov et al., 2019; Gordon et al., 2020).

### The Inactivation of I_K(DR)_ Modified by RDV

**Figure 4:**
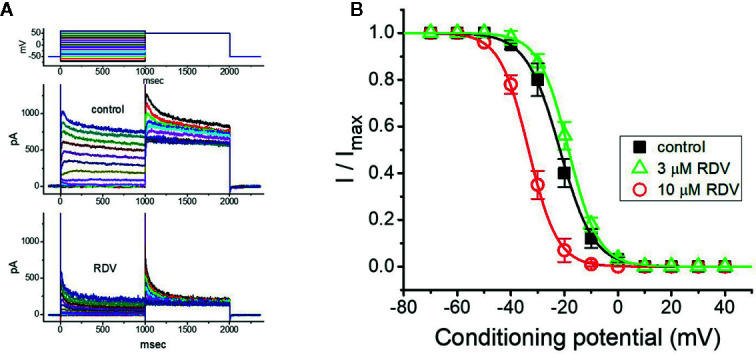
Effect of RDV on the steady-state inactivation curve of *I*_K(DR)_ in GH_3_ cells. This set of experiments was undertaken with a two-step voltage protocol (as indicated in the uppermost part of **(A)**. **(A)** Representative *I*_K(DR)_ traces obtained in the absence (upper) and presence (lower) of 10 μM RDV. The voltage protocol applied is illustrated in the uppermost part. **(B)** Steady-state inactivation curve of *I*_K(DR)_ in the control (■) and during exposure to 3 μM RDV (**△**) or 10 μM RDV (**○**) (mean ± SEM; n=8 for each point). Each curve noticeably overlaid on the data was fitted by Boltzmann equation detailed in **Materials and Methods**.

As cells were exposed to different RDZ concentrations, the *I*_K(DR)_ in response to membrane depolarization noticeably exhibited an evident peak followed by an exponential decline to a steady-state level. Hence, we further explored the quasi-steady-state inactivation curve of *I*_K(DR)_ attained in the absence or presence of RDV by using a two-step voltage protocol. In this series of experiments, we immersed cells in Tyrode’s solution (Ca^2+^-free), and then filled the electrode with K^+^-containing solution, during electrical recordings. Once whole-cell configuration has been tightly established, we applied a two-pulse protocol, under analog-to-digital conversion, to the examined cells in which different RDV concentrations were present. From the least-squares minimization, the inactivation parameters of *I*_K(DR)_ were appropriately derived in the presence of 3 or 10 μM RDV. As illustrated in **Figures 4A, B**, we constructed the normalized strength of *I*_K(DR)_ (i.e., *I*/*I*_max_) against the conditioning command potentials, and the continuous sigmoidal curve was well fitted with a modified Boltzmann function elaborated under *Materials and Methods*. In the presence of 3 μM RDZ, *V*_1/2_ = −33.4 ± 1.8 mV, *q* = 4.7 ± 0.3 *e* (n = 8), whereas in the presence of 10 μM RDZ, *V*_1/2_ = −18.5 ± 1.7 mV, *q* = 4.5 ± 0.3 *e* (n = 8). Observations from this set of experiments disclosed that during GH_3_-cell exposure to different RDV concentrations, the *V*_1/2_ value of *I*_K(DR)_ inactivation curve attained from these cells could be measurably altered, although modification in the gating charge was not noticed.

### RDV on the Recovery of I_K(DR)_ Blockage Identified in GH_3_ Cells

**Figure 5:**
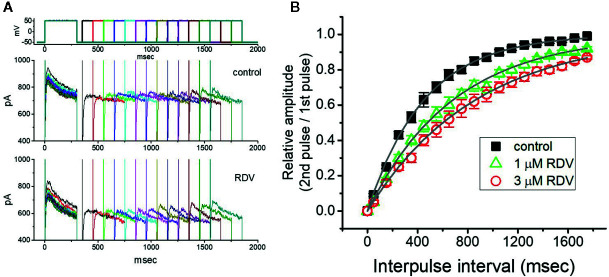
Recovery from *I*_K(DR)_ block produced by RDV. In this set of whole-cell recording experiments, GH_3_ cells, bathed in Ca^2+^-free, Tyrode’s solution, were depolarized from −50 to +50 mV with a duration of 300 ms and different interpulse durations were thereafter applied. **(A)** Superimposed *I*_K(DR)_ traces in the absence (upper) and presence (lower) of 1 μM RDV. Voltage protocol used is denoted in the uppermost part of (A). **(B)** Time course of recovery from *I*_K(DR)_ inactivation achieved in the control (■) and during exposure to 1 μM RDV (**△**) or 3 μM RDV (**○**). The recovery time course in the control, during exposure to 1 μM RDV, and that to 3 μM RDV was satisfactorily fitted to a single exponential with a time constant of 453, 687, and 867 ms, respectively. Each point in this Figure is the mean ± SEM (n=7 for each point).

Recovery from block by RDV was additionally undertaken with another two-step voltage-clamp protocol which comprises an initial (i.e., the first conditioning) depolarizing pulse sufficiently long to allow block to reach block to reach a steady-state level. The membrane voltage was thereafter stepped to +50 mV from −50 mV for a variable time, after a second depolarizing pulse (test pulse) was applied at the same potential as the conditioning pulse (**Figure 5A**). The ratios (2^nd^ pulse/1^st^ pulse) of the peak amplitude of *I*_K(DR)_ evoked in response to the test and the conditioning pulse were employed for a measure of recovery from block, and the values were constructed and then plotted versus interpulse interval (**Figure 5B**). The time course for the recovery of *I*_K(DR)_ block with or without RDV addition was noticed to be described by a single-exponential function. The time constant for current recovery from inactivation in the control was measured to be 453 ± 17 ms (n = 7), whereas the addition of 1 or 3 μM RDV to the examined cells prolonged the time constant to 687 ± 23 (n = 7, *P*<0.05) or 867 ± 37 ms (n = 7, *P*<0.05), respectively. These observations prompted us to indicate that the slowing of recovery caused by adding RDV might be principally owed to the block in open or inactivated state.

### RDV on M-type K^+^ Current (I_K(M)_) in GH_3_ Cells

**Figure 6:**
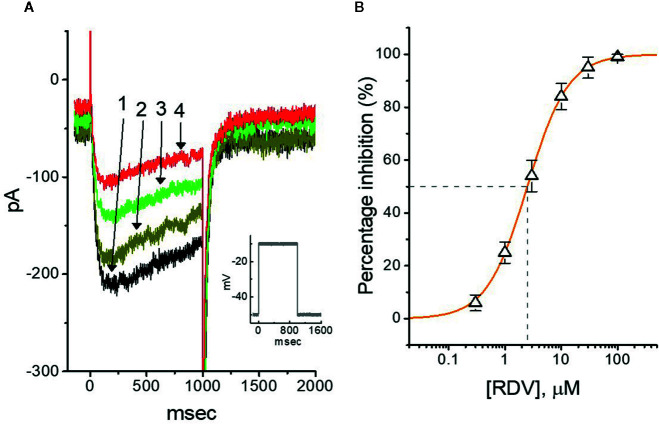
Effect of RDV on M-type K^+^ current (*I*_K(M)_) in GH_3_ cells. The experiments were conducted in cells immersed in high-K^+^, Ca^2+^-free solution and the pipette used was filled with K^+^-containing solution. **(A)** Representative *I*_K(M)_ traces elicited by 1-sec membrane depolarization from −50 to −10 mV (indicated in the Inset). Current trace labeled 1 is control and that labeled 2, 3 or 4 was obtained after the addition of 0.3 μM RDV, 1 μM RDV or 3 μM RDV, respectively. **(B)** Concentration-dependent relation of RDV effect on *I*_K(M)_ amplitude in GH_3_ cells (mean ± SEM; n=9 for each point). The continuous line was accordingly fitted by a Hill function as described under **Materials and Methods**. The IC_50_ value (as indicated in the vertical dashed line) needed for RDV-induced depression of *I*_K(M)_ was identified to be 2.5 μM.

In another separate measurements, we further checked whether the effect of RDV on the amplitude or gating of another type of K^+^ current (i.e., M-type K^+^ current [*I*_K(M)_]) endogenously in GH_3_ cells (Sankaranarayanan and Simasko, 1996; Selyanko et al., 1999; Yang et al., 2019). The cells were bathed in high-K^+^, Ca^2+^-free solution, and the K^+^-containing solution was used to fill up the recording electrode. Of notice, within 1 min of RDV exposure, the *I*_K(M)_ strength of GH_3_ cells was considerably declined (**Figure 6A**). For example, at as the cells were depolarized from −50 to −10 mV, the addition of 3 μM RDV decreased *I*_K(M)_ amplitude from 176 ± 25 to 78 ± 19 pA (n=9, *P*<0.05), and after removal of RDV, current amplitude returned to 169 ± 24 pA (n=9). We consequently constructed the association between the RDV concentration and the degree of *I*_K(M)_ suppression. The half-maximal concentration (i.e., IC_50_) needed for depressant effect of RDV on *I*_K(M)_ was yielded to be 2.5 μM, and at a concentration of 100 μM, it nearly fully depressed current strength (**Figure 6B**). It is apparent, therefore, that RDV can exert a pronounced action on the inhibition of *I*_K(M)_ identified in GH_3_ cells.

### Effect of RDV on I_K(M)_ Triggered by Triangular Ramp Pulse With Varying Durations

**Figure 7:**
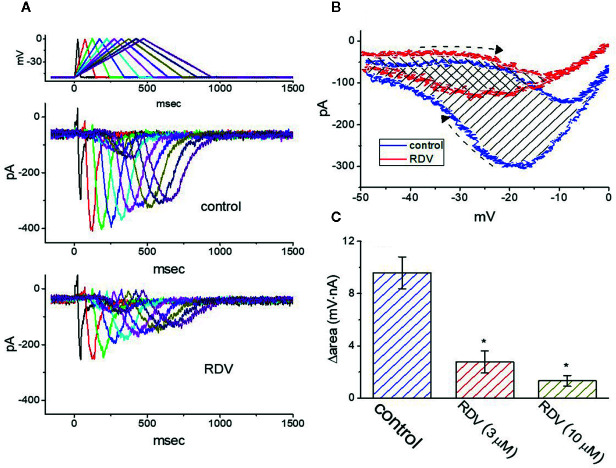
Effect of RDV on *I*_K(M)_ in response to isosceles-triangular ramp pulse with different durations (40–940 ms) which was particularly designed to mimic different depolarizing and repolarizing slope of bursting pattern. **(A)** Superimposed *I*_K(M)_ traces in response to the uppermost voltage protocol obtained in the absence (upper) and presence (lower) of 3 μM RDV. The uppermost part is the voltage profile delivered. **(B)** Effect of RDV (3 μM) on voltage dependent hysteresis (i.e., the relationship of forward and reverse current versus membrane voltage) of *I*_K(M)_ elicited by triangular ramp pulse with a duration of 940 ms. Blue or red current trajectory indicates the absence or presence of 3 μM RDV, respectively. Dashed arrows indicate the direction of *I*_K(M)_ in which time passes during the elicitation by 940-ms triangular ramp pulse. **(C)** Summary bar graph showing the effect of RDV on the Δarea (as indicated in shaded area in **(B)** (mean ± SEM; n=9 for each bar). The Δarea with respect to the voltage-dependent hysteresis of *I*_K(M)_ taken with or without 3 μM RDV addition is indicated as shaded area in **(B)**. ^*^Significantly different from control (*P*<0.05).

Previous experiments disclosed the capability of *I*_K(M)_ strength to modulate the patterns of bursting firing in central neurons (Brown and Passmore, 2009). Therefore, we wanted to evaluate how RDV could have any propensity to perturb *I*_K(M)_ responding to long-lasting triangular ramp pulse with varying durations, which were achieved by digital-to-analog conversion. In the presence experiments, the examined cell was voltage-clamped at −50 mV and the upsloping (forward) limb from −50 to 0 mV followed by the downsloping (backward) limb back to −50 mV with varying durations (40–940 ms) was thereafter applied. As demonstrated in **Figure 7A**, once the slope of ramp pulse was declined, the maximal strength of *I*_K(M)_ triggered by the upsloping limb of triangular ramp pulse was progressively raised, whereas the peak amplitude of *I*_K(M)_ was initially elevated and followed by gradual decline. However, once 3 μM RDV was added, the strength of the current responding to both rising and falling ramp pulse was noticeably decreased (**Figure 7A**). For instance, as the duration of triangular ramp pulse applied was set at 940 ms (i.e., slope= ± 0.1 V/sec), the addition of 3 μM RDV decreased current amplitude measured at the upsloping or downsloping limbs from 150 ± 12± to 83 ± 9 pA (n=8, *P*<0.05), or from 294 ± 23 to 131 ± 11 pA (n=8, *P*<0.05). The experimental results illustrated that the strength of *I*_K(M)_ in the upsloping lime was considerably raised as the duration of triangular ramp pulse elevated, while that in the downsloping limb was gradually declined, and that adding RDV contributed to a decline of *I*_K(M)_ by a time-dependent manner in GH_3_ cells.

The voltage hysteresis of ionic currents has been demonstrated to have an impact on electrical behaviors of action-potential firing (Männikko et al., 2005; Fürst and D’Avanzo, 2015; Hsu et al., 2020). The *I*_K(M)_ amplitude triggered by the upsloping limb of triangular voltage ramp was considerable lower that that by the downsloping limb, strongly indicating a voltage-dependent hysteresis for *I*_K(M)_ as depicted in **Figure 7B**, according to the relationship of *I*_K(M)_ versus membrane voltage. As the duration of triangular pulse raised from 40 to 940 ms (i.e., the slope became decreased), the hysteresis degree for *I*_K(M)_ was decreased. Of notice, by adding RDV (3 μM), *I*_K(M)_ evoked in the upsloping limb of long-lasting triangular ramp decreased to a less extent than which measured from the downsloping ramp. For instance, in controls (i.e., RDV was not present), *I*_K(M)_ at the level of −20 mV elicited upon the upsloping and downsloping ends of triangular ramp pulse were 78 ± 9 and 301 ± 23 pA (n=8), respectively, the values of which were noticed to differ significantly between them (*P*<0.05). Furthermore, by adding 3 μM RDV, the strength of forward and backward *I*_K(M)_ at the same membrane voltage was evidently declined to 65 ± 6 and 135 ± 18 pA. Therefore, the strength of RDV-induced current inhibition at the upsloping (forward) and downsloping (reverse) limbs of triangular ramp differ significantly. The addition of 3 μM RDV decreased *I*_K(M)_ amplitude evoked at the upsloping or downsloping limb of triangular ramp pulse by about 17% or 55%, respectively.

As described by the dashed arrows in **Figure 7B**, upon the difference (i.e., Δarea) in area under the curve in the forward (upsloping) and backward (downsloping) direction, furthermore, we quantified the degree of voltage-dependent hysteresis of *I*_K(M)_. It showed that the amount of voltage hysteresis responding to 940-ms triangular ramp pulse was considerably lessened in the presence of RDV. **Figure 7C** summarized the data demonstrating the effects of RDV (3 or 10 μM) on the area under such curve. For instance, in addition to its depression of *I*_K(M)_ amplitude, the presence of 3 μM RDV decreased the area responding to long-lasting triangular ramp, as illustrated by a specific reduction of Δarea from 9.6 ± 1.2 to 2.8 ± 0.8 mV·nA.

### Mild Inhibition by RDV of erg-Mediated K^+^ Current (I_K(erg)_) in GH_3_ Cells

**Figure 8:**
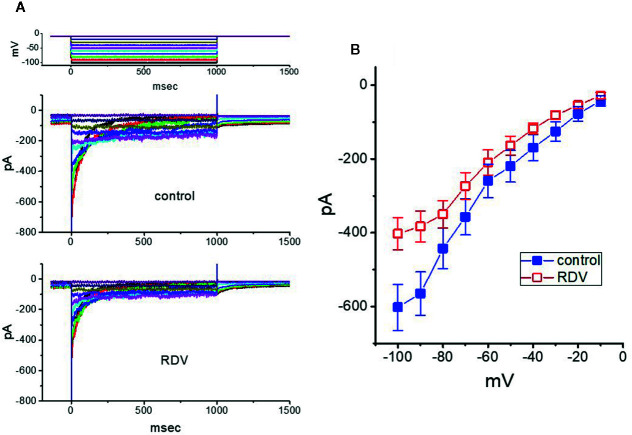
Effect of RDV on *erg*-mediated K^+^ current (*I*_K(erg)_) enriched in GH_3_ cells. The experiments were undertaken in cells bathed in high-K^+^, Ca^2+^-free solution, and we filled the electrode using K^+^-containing solution. **(A)** Superimposed *I*_K(erg)_ traces elicited by a series of voltage pulse as indicated in the uppermost part of **(A)**. The traces in the upper part are controls (i.e., RDV was not present), and those in the lower part was obtained 2 min after application of 30 μM RDV. **(B)** Averaged *I-V* relationships of *I*_K(erg)_ obtained in the absence (**■**) and presence (**□**) of 30 μM RDV (mean ± SEM; n=9 for each point). Current amplitude was obtained at the beginning of each hyperpolarizing command pulse.

Further, we investigated the potential modifications of RDV on another K^+^ current (i.e., *I*_K(erg)_) also endogenously in these cells. Under our experimental conditions, the deactivating inwardly directed *I*_K(erg)_ could be robustly elicited from −10 mV holding potential to a range of voltage pulses from −100 to −10 mV within 1 sec (Wu et al., 2000; Huang et al., 2011; Hsu et al., 2020). When GH_3_ cells were exposed to RDV at a concentration of 30 μM, the amplitude of deactivating *I*_K(erg)_ was mildly but significantly depressed throughout the entire voltage-clamp pulses applied (**Figure 8**). For example, at the level of −90 mV, the peak amplitude of *I*_K(erg)_ was noticeably decreased from 565 ± 59 to 383 ± 42 pA (n=9, *P*<0.05), as cells were exposed to 30 μM RDV. After the agent was washed out, the strength was back to 554 ± 51 pA (n=8). Alternatively, adding 30 μM RDV lessened whole-cell conductance of peak *I*_K(erg)_ measured between −50 and −90 mV from 8.7 ± 0.8 to 5.8 ± 0.7 nS. Therefore, as compared with *I*_K(DR)_ or *I*_K(M)_, the *I*_K(erg)_ in these cells is relatively resistant to being blocked by RDV. However, the RDV effect on *I*_K(erg)_ tends to be rapid in onset, and it should be independent of its perturbing effect on the activity of RNA-polymerase.

### Stimulation by RDV of I_MEP_ in GH_3_ Cells

**Figure 9:**
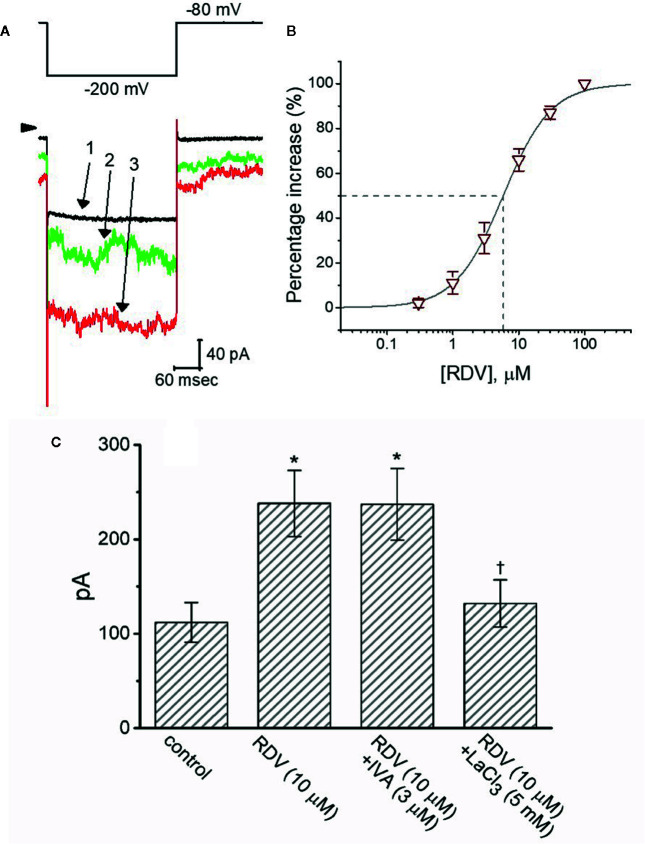
The stimulatory effect of RDV on membrane electroporation-induced current (*I*_MEP_) identified in GH_3_ cells. In this separate set of experiments, we bathed cells in Ca^2+^-free, Tyrode’s solution and the recording pipette was filled with K^+^-containing solution. **(A)** Representative *I*_MEP_ traces obtained in the control (**1**) and during cell exposure to 3 μM RDV (**2**) or 10 μM RDV (**3**). The voltage-clamp protocol applied is denoted in the upper part, arrowhead is the zero current level and the calibration mark at the right lower part applies all current traces. Noticeably, the addition of RDV causes a measurable increase in the amplitude of *I*_MEP_ elicited by large membrane hyperpolarization from −80 to −200 mV with a duration of 300 ms. **(B)** Concentration-dependent stimulation of *I*_MEP_ produced by RDV in GH_3_ cells (mean ± SEM; n=8 for each point). Current amplitude was measured at the end of hyperpolarizing pulse from −80 to −200 mV with a duration of 300 ms, and the vertical dashed line is placed at the IC_50_ value required for RDV-stimulated *I*_MEP_. **(C)** Summary bar graph showing effect of RDV, RDV plus ivabradine (IVA) and RDV plus LaCl_3_ on *I*_MEP_ amplitude (mean ± SEM; n=8 for each bar). Current amplitude was taken at the end of hyperpolarizing voltage pulse from −80 to −200 mV with a duration of 300 ms. ^*^Significantly different from control (*P*<0.05) and ^Ɨ^significantly different from ZDV (10 M) alone group (*P*<0.05).

It has been reported that *I*_MEP_ elicited in response to large membrane hyperpolarization (Dyachok et al., 2010; Liu et al., 2012; Wu et al., 2012; So et al., 2013; Chiang et al., 2014; Chang et al., 2020a). To study whether RDV possibly perturb this type of ionic current, we bathed cells in Tyrode’s solution (Ca^2+^-free) and performed whole-cell current recordings. As described in previous observations (Dyachok et al., 2010; Wu et al., 2012; Chang et al., 2020a; Chang et al., 2020b), when the cell was voltage-clamped at −80 mV and the 300-ms hyperpolarizing pulse to −200 mV was applied to evoke *I*_MEP_. As depicted in **Figures 9A, B**, when cells were continually exposed to RDV, the amplitude of *I*_MEP_ elicited by such large hyperpolarization was progressively raised. For instance, 3 μM RDV conceivably elevated *I*_MEP_ amplitude from 112 ± 21 to 238 ± 35 pA (n=8, *P*<0.05) at the level of −200 mV. After washout, current amplitude was back to 124 ± 24 pA (n=8). Additionally, as K^+^ ions in the internal solutions were replaced with equimolar concentrations of NMDG^+^, this current could still be enhanced through adding 3 μM RDV; however, current magnitude tended to be smaller. **Figure 9B** shows the association between the concentration of RDV and the degree of *I*_MEP_ increase. RDV could concentration-dependently elevate the amplitude of *I*_MEP_ activated during large step hyperpolarization. The half-maximal concentration (EC_50_) needed for the stimulatory effect of RDV on *I*_MEP_ was noticed to be 5.8 μM. Our findings disclosed the effectiveness of RDV in generating a stimulatory action on *I*_MEP_ in GH_3_ cells. **Figure 9C** depicts summary bar graph showing the effect of RDV, RDV plus ivabradine or RDV plus LaCl_3_ on *I*_MEP_. The results indicate that RDV-stimulated *I*_MEP_ was overcome by subsequent addition of LaCl_3_ (5 mM), but not by ivabradine (3 μM). Ivabradine or hydroxychloroquine was demonstrated to be an inhibitor of hyperpolarization-activated cation current (Capel et al., 2015; Hsiao et al., 2019). Subsequent addition of chlorotoxin (1 μM), a blocker of Cl^-^ channels, was unable to reverse RDV-induced *I*_MEP_ (242 ± 38 pA [in the presence of 3 μM RDV] versus 239 ± 41 pA [in the presence of 3 μM RDV plus 1 μM chlorotoxin]; n=8, *P*>0.05). In consequence, the RDV-stimulated *I*_MEP_ identified in GH_3_ cells is unlikely to result from its activation of hyperpolarization-activated cation current.

## Discussion

In this study, we noticed that in a time- and concentration-dependent fashion the presence of RDV depressed the strength of delayed-rectifier K^+^ current (*I*_K(DR)_) in pituitary tumor (GH_3_) cells. The rate of current inactivation apparently became fastened as the RDV concentration increased. In another perspective, the suppression of RDV on *I*_K(DR)_ is evidently associated with an increasing inactivation rate of the current responding to membrane depolarization. Specifically, the relative block of *I*_K(DR)_ induced by the RDV concentrations could be hence fitted in an exponential fashion. From the minimal reaction scheme (as shown in **Supplementary Material (1)**), the value of dissociation constant (*K*_D_) required for RDV-induced block of *I*_K(DR)_ in GH_3_ cells was yielded to be 3.04 μM, which is close to effective IC_50_ value (2.8 μM) for RDV-mediated inhibition of late *I*_K(DR)_, but is lower than that (10.1 μM) for its block of initial peak *I*_K(DR)_.

Alternatively, during cell exposure to different RDV concentrations, the inactivation parameter (i.e., *V*_/12_ value) for the inactivation curve of *I*_K(DR)_ emerging from GH_3_ cells can be evidently adjusted, with no modifications of the gating charge. The presence of RDV (1 and 3 μM) induced *I*_K(DR)_ block from the inactivation could be also noticeably recovered with single exponential of 687 and 867 ms, respectively. In this scenario, the present observations disclose that the RDV molecules tend to accelerate *I*_K(DR)_ inactivation in a concentration- and state-dependent fashion, implying that they reach the blocking site of the channel, only when the channel involved resides in the open conformational state. The EC_50_ value of RDV against SARS-CoV-2 existing in Vero E6 cells was noticeably measured to be 1.76 μM, indicating that its working concentration is more than likely achieved *in vivo* (Wang et al., 2020). In the present study, the RDV presence was also observed to inhibit *I*_K(DR)_ in Jurkat T-lymphocytes in a time- and concentration-dependent fashion (**Supplementary Material (2)** and **Supplementary Figure 1**). Besides its antiviral activity, similar to chloroquine, RDV per se might to some extent effect an immune-modulating activity possibly through the inhibition of K_V_ channels.

The current observations pointed out that with effective IC_50_ of 2.5 μM in GH_3_ cells, RDV was capable of depressing the strength of *I*_K(M)_. Moreover, the voltage-dependent hysteretic changes of ionic currents are hypothesized to play an essential characteristic in the behaviors of different types of electrically excitable cells. In the current study, echoing previous observations (Männikko et al., 2005; Fürst and D’Avanzo, 2015; Hsu et al., 2020), the *I*_K(M)_ endogenously in GH_3_ cells was also observed to go either through a voltage-dependent hysteresis, or a mode-shift in the conditions of which the voltage sensitivity of gating charge movements is dependent on the previous state. By long-lasting triangular ramp pulse, RDZ noticeably suppressed the strength of voltage-dependent hysteresis for *I*_K(M)_ elicitation. As such, we provide the experimental results strongly demonstrating that there is a perturbing effect of RDZ on such non-equilibrium property in M-type K^+^ channels in electrically excitable cells such as GH_3_ cells, although how RDZ-induced changes in voltage hysteresis of *I*_K(M)_ are connected with the behaviors of electrically excitable cells is unclear.

The present study discloses that RDV can directly inhibit *I*_K(M)_ and *I*_K(DR)_ in pituitary GH_3_ cells, suggesting that this compound per se presumably is not an inactive prodrug. The depression of these K^+^ currents would be expected to be potentially charged with its actions on activities in various types of cell including GH_3_ cells. A current report noticeably demonstrated the occurrence of hypokalemia present in the patients with coronavirus disease 2019 (Chen et al., 2020). It is reasonable to presume that, apart from its effects on the viral polymerase and the proofreading exoribonuclease (Agostini et al., 2018; Brown et al., 2019; Tchesnokov et al., 2019; Gordon et al., 2020), to what extent RDV-induced perturbations of ion channels unexpectedly identified in this study participates in its antiviral actions has yet to be further delineated.

Our results are in accordance with previous findings demonstrating that the large hyperpolarization induced inward currents (i.e., *I*_MEP_) occur in glioma cells, heart cells, pituitary cells, and macrophages (Dyachok et al., 2010; Liu et al., 2012; So et al., 2013; Chiang et al., 2014; Chang et al., 2020a; Chang et al., 2020b). Such hyperpolarization-induced activation followed by irregular time course indicates that *I*_MEP_ was produced by transient rupture of cell membrane caused by the electrical field tied to large hyperpolarization (Dyachok et al., 2010; Wu et al., 2012; So et al., 2013; Chang et al., 2020a; Chang et al., 2020b). In the current study, the presence of RDV was effective at increasing *I*_MEP_ dose-dependently with EC_50_ value of 5.8 μM. Further addition of LaCl_3_, yet not that of chlorotoxin or ivabradine, was noticed to reverse RDV-stimulated *I*_MEP_. Previous observations have reported the effectiveness of AUY922, a small-molecule inhibitor of heat-shock protein 90 (HSP90), in stimulating *I*_MEP_ in glioblastoma cells through a mechanism independent of HSP90 inhibition (Chiang et al., 2014). As a corollary, stimulation by RDV of *I*_MEP_ in GH_3_ cells also tends to be direct and is unlikely to be mediated through a mechanism linked to its prevailing actions on RNA polymerases.

The MEP-perturbed portion of the surface membrane can initiate ion fluxes into and out of the cell, hence producing a massive change in the ionic milieu of the cytosol. This effect has applications in biotechnology and medicine and, hence, has been the subject of both experimental and theoretical work (Gehl, 2003; So et al., 2013; Napotnik and Miklavčič, 2018). Due to high conductance of MEP-induced channels, even at low probability that would be open, significant currents have the propensity to flow, thereby altering the electrical behavior of cells (Vernier et al., 2009; Kaminska et al., 2012). Alternatively, previous studies have shown that the activity of MEP-elicited channels could act as a component of trans-plasma membrane electron transport, to which the targeting of mitochondrial permeability transition pore (mPTP) is closely linked (Del Principe et al., 2011; Bagkos et al., 2015). Therefore, whether RDV-stimulated perturbations of *I*_MEP_ in different types of cells can account for its antiviral effectiveness is worth further investigation.

**Figure 10:**
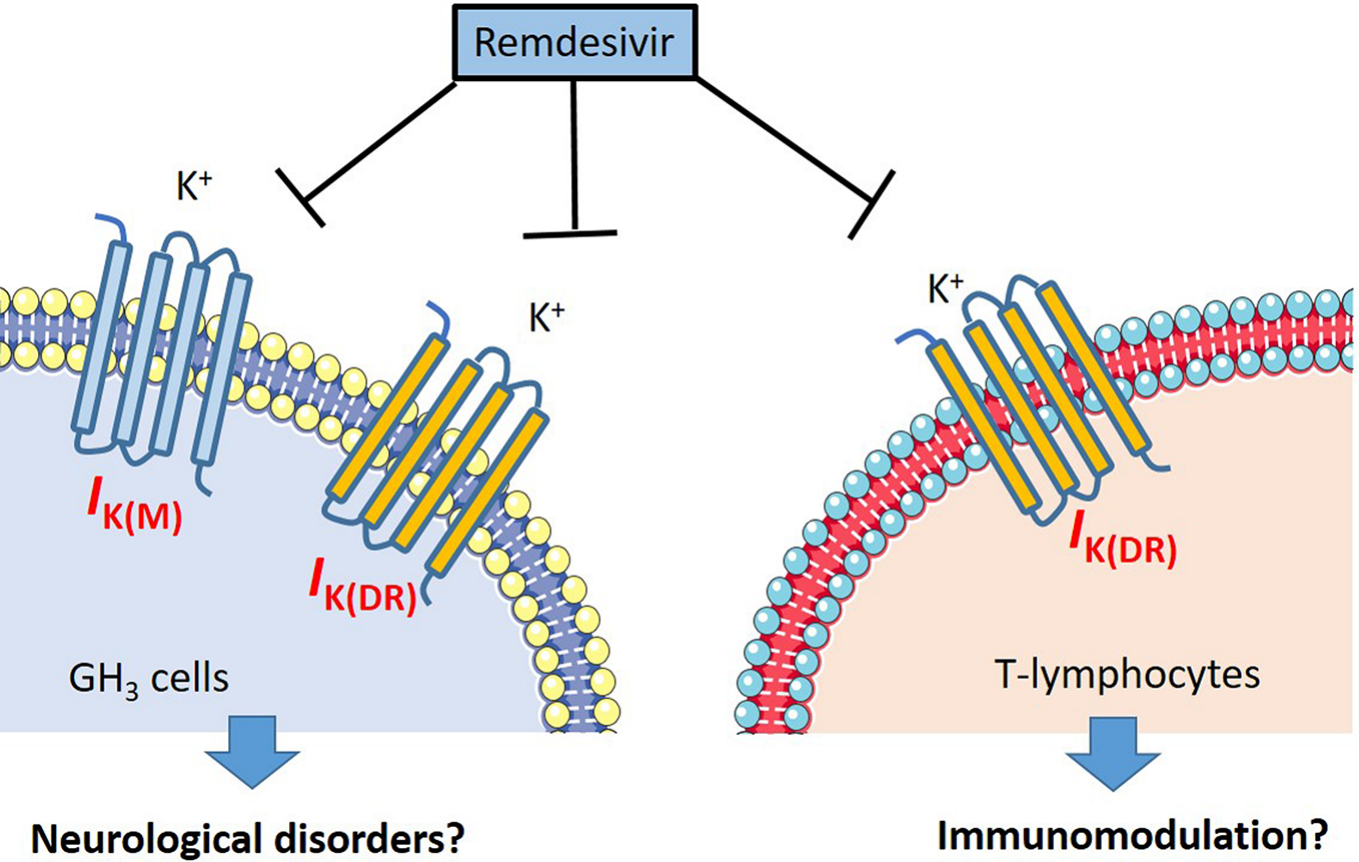
The illustration of possible mechanism regarding the RDV induced perturbations on neurons and lymphocytes.

Aconitine, a material agent with potential cardiotoxicity, has been described to modify the gating of *I*_K(DR)_ in lymphocytes, neural, and cardiac cells (Lin et al., 2008). Aconite alkaloids from *Aconitum carmichaelii* were recently demonstrated to exert antiviral activity against cucumber mosaic virus (Xu et al., 2019). Additionally, curcuminoids have been demonstrated to depress *I*_K(DR)_ as well as to fasten *I*_K(DR)_ inactivation in insulin-secreting cells (Kuo et al., 2018), as well as to possess potent antiviral activities against coronavirus (Wen et al., 2007). Though additional experiments are required to verify the current results, RDV-induced effects on ionic currents demonstrated could be a confounding factor and the notable ionic mechanism underlying its modifications on cell behaviors occurring *in vitro* or *in vivo*. The summary of our findings regarding the possible perturbations of RDV is illustrated in **Figure 10**.

RDV-perturbed suppression of *I*_K(DR)_ or *I*_K(M)_ demonstrated is independent of its possible actions on RNA polymerase (Agostini et al., 2018; Brown et al., 2019; Gordon et al., 2020). in another perspective, it is intriguing to investigate whether the modification by RDV of RNA polymerase would attribute to its blocking of membrane *I*_K(DR)_ or *I*_K(M)_, as well as from its stimulation of *I*_MEP_ in different cell types. To what extent RDV-induced perturbations on membrane ionic currents confers its effectiveness in antiviral activities thus remains to be resolved. Following intravenous administration of RDV can readily pass across the blood-brain barriers (Warren et al., 2016; Ferren et al., 2019; Lucey, 2019). Recent studies have demonstrated that CoVs might exert neuro-invasive potential (Ferren et al., 2019; Li H. et al., 2020). Findings from the present observations might shed the light to the notion that the effect of RDV on the gating of the currents are intimately tied to its antiviral actions or variable forms of neurological effects (Ferren et al., 2019); however, the present observations do not preclude the further investigations and uses of RDV in the treatment of SARS-CoV-2 infection.

## Data Availability Statement

The raw data supporting the conclusions of this article will be made available by the authors, without undue reservation.

## Author Contributions

S-NW designed the experiments. Z-HG, S-WL, W-KL, and S-NW carried out the experiments. P-YL provided the resources. W-TC and S-NW analyzed the data. W-TC and S-NW wrote the paper. All authors contributed to the article and approved the submitted version.

## Funding

This study was financially supported by the grants from Ministry of Science and Technology (MOST-108-2314-B-006-094) and National Cheng Kung University (NCKUH-10709001 and D107-F2519), Taiwan. The funders are not involved in the study design, data collection, analyses, or interpretation.

## Conflict of Interest

The authors declare that the research was conducted in the absence of any commercial or financial relationships that could be construed as a potential conflict of interest.

## Abbreviations

AMPCPP, α,β-methylene-ATP; ATP, adenosine triphosphate; CoV, coronavirus; DPCPX, 8-cyclopentyl-1,3-dipropylxanthine; EC_50_, concentration required for half-maximal stimulation; IC_50_, concentration required for half-maximal inhibition; I-V, current versus voltage; MEP, membrane electroporation; I_K(DR)_, delayed-rectifier K^+^ current; I_MEP_, membrane electroporation-induced current; I_K(erg)_, erg-mediated K^+^ current; K_D_, dissociation constant; NMDG^+^, N-methyl-D-glucamine^+^; RDV, remdesivir (GS-5734); SEM, standard error of mean; τ, time constant; K_V_ channel, voltage-gated K^+^ channel; I_K(M)_, M-type K^+^ current.
