# Supplementary material for: Evidence for the Effectiveness of Remdesivir (GS-5734), a Nucleoside-Analog Antiviral Drug in the Inhibition of I K(M) or I K(DR) and in the Stimulation of I MEP
Source: Front Pharmacol. 2020 Jul 21;11:1091. doi: 10.3389/fphar.2020.01091 (PMC7385287; doi:10.3389/fphar.2020.01091)
Supplement: Supplementary file 1 [file DataSheet_1.pdf]

Supplementary material (1)

Data recordings and analyses

The signals, consisting of potential and current traces, were stored online on an ASUS VivoBook Flip-14 touchscreen laptop computer (TP412U; Taipei, Taiwan) at 10 kHz through a Digidata 1440A interface (Molecular Devices). During the recordings, data acquisition system connected with the latter device was operated by pCLAMP 10.7 (Molecular Devices). Current signals were low-pass filtered at 3 kHz with an FL-4 four-pole Bessel filter (Dagan, Minneapolis, MN). We analyzed the signals offline by using either pCLAMP 10.7 (Molecular Devices), OriginPro (OriginLab; Scientific Formosa, Taipei, Taiwan) or different custom-made macros created from Microsoft Excel^{TM} 2019.

To estimate the percentage inhibition of RDV on $I_{K(DR)}$, we bathed cells in Ca^{2+}-free, Tyrode's solution (i.e., 5.4 mM K^+-containing solution), each cell was depolarized for 1 sec from a holding potential of -50 to +50 mV, and the amplitude of $I_{K(DR)}$ during the exposure to different RDV concentrations was compared with the control value. To determine that of RDV on $I_{K(M)}$, we bathed cells in high-K^+, Ca^{2+}-free solution and the examined cell was depolarized from -50 to -10 mV. We appropriately fitted the concentration-dependent effect of RDV on the inhibition of $I_{K(DR)}$ (i.e., initial peak and sustained [late] currents) with a Hill function by using a nonlinear least-square fitting algorithm. That is,

$$\text{Percentage inhibition (\%)} = \frac{[\text{RDV}]^{n_H} \times E_{max}}{[\text{RDV}]^{n_H} + IC_{50}^{n_H}}$$

where [RDV] is the RDV (remdesivir) concentration; E_{max} is the RDV-induced maximal inhibition of either initial peak and sustained $I_{K(DR)}$ or $I_{K(M)}$; and IC_{50} and n_H are the concentration required for a 50% inhibition and the Hill coefficient, respectively.

The concentration-response data for RDV-induced stimulation of I_{MEP} emerging from

GH_3 cells were created and least-squares fitted with a modified Hill function:

$$y = \frac{[\mathrm{RDV}]^{n_H} \times E_{max}}{[\mathrm{RDV}]^{n_H} + EC_{50}^{n_H}}$$

where y is the relative amplitude of I_{MEP}, [RDV] is the RDV (remdesivir) concentration given, the EC_{50} is the concentration required for a 50% increase in the current amplitude, and E_{max} is the maximal increase in I_{MEP} produced by 100 μM RDV.

To determine the steady-state inactivation curve of $I_{K(DR)}$ in the absence and presence of different RDV concentrations, we collected the results and then constructed the relationships between the normalized amplitude of $I_{K(DR)}$ and the conditioning potentials applied. The data achieved were least-squares fitted with a Boltzmann function of the following form:

$$\frac{I}{I_{max}} = \frac{1}{\left\{1 + exp\left[\frac{(V - V_{1/2})qF}{RT}\right]\right\}}$$

where I_{max} in the maximal amplitude of $I_{K(DR)}$ measured at the level of +50 mV; $V_{1/2}$ the voltage (in mV) at which half-maximal inhibition occurs; q the apparent gating charge (in elementary charge [e]) of the inactivation curve; F the Faraday constant; R the universal gas constant; and T the absolute temperature (in Kelvin).

Kinetic study of remdesivir- (RDV)-induced block of $I_{K(DR)}$ in pituitary tumor (GH_3) cells

To provide quantitative estimate for RDV-induced block of $I_{K(DR)}$, we further analyzed the time-dependent trajectories for the relative block of $I_{K(DR)}$ (i.e., $(I_{control}$-$I_{RDV})/I_{control}$) observed in these cells. We fitted the time courses of relative block in the presence of different RDV concentrations by a single-exponential function (**Fig. 1C and 1D**). The concentration dependence of relative block of $I_{K(DR)}$ in response to long-lasting step

depolarization was shown in **Fig. 1C and 1D**. The results demonstrated that the exposure of cells to RDV produced a concentration-dependent raise in the rate ($1/\tau$) of relative block of $I_{K(DR)}$.

The inhibitory action of RDV on depolarization-elicited $I_{K(DR)}$ measured from GH_3 cells is satisfactorily explained by state-dependent block that preferentially binds to the open state of the channel. A minimal reaction scheme was derived as the following:

$$C \underset{\beta}{\overset{\alpha}{\rightleftarrows}} O \underset{k_{-1}}{\overset{k_{+1}^{*}\cdot[RDV]}{\rightleftarrows}} O\cdot RDV$$

where [RDV] is the remdesivir (RDV) concentration; α or β is the voltage-gated rate constant for the opening or closing of K_V channels, respectively; k_{+1}^{*} or k_{-1} is the blocking (i.e., on) or unblocking (i.e., off) rate constant produced by the presence of RDV, respectively; and C, O, or O·RDV shown in the scheme represents the closed (resting), open, or open-blocked states, respectively.

The blocking (i.e., on) and unblocking (i.e., off) rate constants, namely k_{+1}^{*} and k_{-1}, were determined from the time constants (τ) of depolarization-evoked relative block (($I_{control}$-I_{RDV})/$I_{control}$) of $I_{K(DR)}$ obtained in different concentrations of RDV. The rate constants ($1/\tau$) obtained were allowed to be computed using the relation (**Fig. 1D**).

$$\frac{1}{\tau} = k_{+1}^{*} \times [RDV] + k_{-1}$$

where k_{+1}^{*} or k_{-1} is acquired from the slope or from the y-axis intercept at [RDV] = 0 of the linear regression in which the reciprocal time constants ($1/\tau$) versus the RDV concentrations were interpolated, and [RDV] is the RDV concentration.

As a consequence, on the basis of the first-order binding scheme elaborated above, the relationship between $1/\tau$ and [RDV] became linear with a correlation coefficient of 0.96 (**Fig.**

1D). The blocking or unblocking rate constants was estimated to be 2.01±0.02 $sec^{-1}\mu M^{-1}$ or 6.12±0.02 sec^{-1} (n=9), respectively; consequently, the results yielded the value of dissociation constant ($K_D = k_{-1}/(k_{+1}^{*}\cdot[RDZ])$) of 3.04 μM.

Supplementary material (2)

Effect of RDZ on delayed-rectifier K^+ current ($I_{K(DR)}$) identified in Jurkat T-lymphocytes

In this separate set of experiments, Jurkat T-lymphocytes were dispersed and then bathed in Ca^{2+}-free, Tyrode's solution and the recording pipette was filled with K^+-containing solution. The Jurkat T cell line is a CD45-deficient clone derived from the E6-1 clone of Jurkat human T cell leukemic cell line, which has been demonstrated to express $K_V1.3$-type $I_{K(DR)}$. As cells were exposed RDZ, the amplitude of $I_{K(DR)}$ elicited by membrane depolarization from -50 to +50 mV with a duration of 300 msec was progressively decreased (**Supplementary Figure 1A**). The averaged current-voltage relationships of $I_{K(DR)}$ in the absence and presence of 3 mM RDV are illustrated in **Supplementary Figure 1B**. For example, at the level of +50 mV, the addition of 3 μM RDV significantly decreased $I_{K(DR)}$ amplitude by 46.6±2.4% from 174±28 to 93±16 pA (n=8, P<0.05). Therefore, the addition of RDZ was effective at depressing $I_{K(DR)}$ in combination with increased rate of current inactivation in these cells.

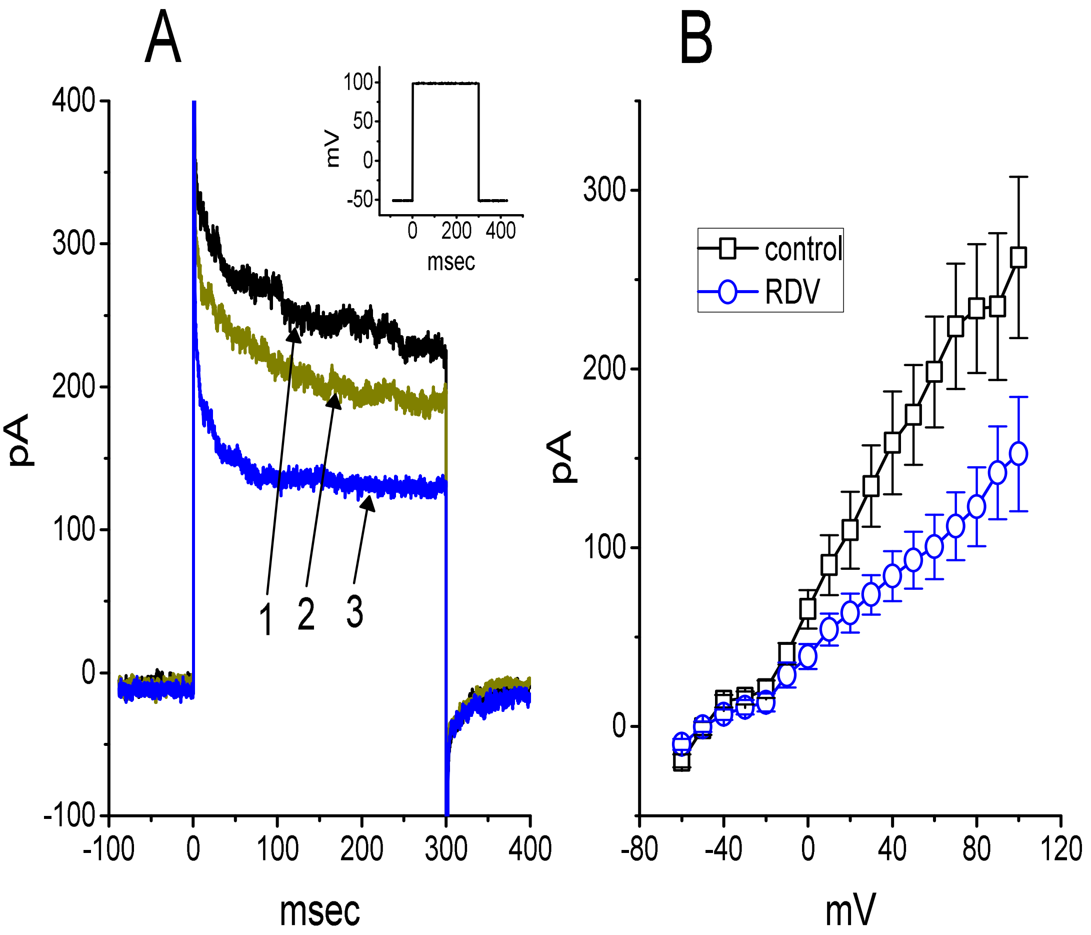

Supplementary Figure 1. Inhibitory effect of RDZ (3 μM) on delayed-rectifier K^+ current ($I_{K(DR)}$) identified in Jurkat T-lymphocytes. **(A)** Superimposed current traces obtained in the control (**1**) and during the exposure to 1 μM RDV (**2**) or 3 μM RDV (**3**). Inset indicate the voltage protocol used. **(B)** Averaged *I-V* relationships of $I_{K(DR)}$ in the absence (□) and presence (○) of 3 μM RDV (mean±SEM; n=8 for each point). Current amplitude was measured at the end of depolarizing pulse from -50 to +50 mV with a duration of 300 msec.
